# Novel R Shiny Tool for Survival Analysis With Time-Varying Covariate in Oncology Studies: Overcoming Biases and Enhancing Collaboration

**DOI:** 10.1200/CCI-25-00225

**Published:** 2026-01-30

**Authors:** Yimei Li, Yang Qiao, Fei Gao, Jordan Gauthier, Qiang (Ed) Zhang, Jenna Voutsinas, Wendy Leisenring, Ted Gooley, Corinne Summers, Alexandre Hirayama, Cameron J. Turtle, Rebecca Gardner, Jarcy Zee, Qian (Vicky) Wu

**Affiliations:** ^1^ Department of Biostatistics, Epidemiology, and Informatics, University of Pennsylvania, Philadelphia, PA; ^2^ Department of Statistics, Iowa State University, Amers, IA; ^3^ Vaccine and Infectious Disease Division, Fred Hutchinson Cancer Center, Seattle, WA; ^4^ Clinical Research Division, Fred Hutchinson Cancer Center, Seattle, WA; ^5^ Wills Eye Hospital, Thomas Jefferson University, Philadelphia, PA; ^6^ Seattle Children's, Seattle, WA; ^7^ The University of Sydney, Camperdown, NSW, Australia; ^8^ Royal North Shore Hospital, Sydney, NSW, Australia; ^9^ Translational Science and Therapeutics, Fred Hutchinson Cancer Center, Seattle, WA; ^10^ St Jude Hospital, Memphis, TN

## Abstract

**PURPOSE:**

Our study is motivated by evaluating the role of hematopoietic cell transplantation (HCT) after chimeric antigen receptor T-cell (CAR-T) therapy for ALL, a debated topic. Because patients may receive HCT at different times after CAR-T infusion or never, HCT post–CAR-T should be considered as a time-varying covariate (TVC).

**METHODS:**

Standard Cox models and Kaplan-Meier (KM) curves (naïve method) assume that TVC status is known and fixed at baseline, which can yield biased estimates. Landmark analysis is a popular alternative but depends on a chosen landmark time. Time-dependent (TD) Cox model is better suited for TVC although visualizing survival curves is complex. The newly proposed Smith-Zee method generates appropriate survival curves from TD Cox models.

**RESULTS:**

To address these challenges, we developed an open-source R Shiny tool integrating multiple models (naïve Cox, landmark Cox, and TD Cox) and curves (naïve KM, landmark KM, Smith-Zee, and Extended KM) to facilitate TVC analysis. Reanalysis of post–CAR-T HCT's effect on leukemia-free survival (LFS) showed consistent results between naïve and TD Cox models, whereas landmark analyses varied by landmark time. A separate data analysis of chronic graft-versus-host disease and survival showed that substantial differences emerged across statistical methods. Simulations revealed increased bias in naïve methods when TVC changed late and minimal bias when TVC changes occurred early relative to time to events.

**CONCLUSION:**

We recommend TD Cox models and Smith-Zee curves for robust TVC analysis. Our R Shiny tool supports standardized analyses without requiring data sharing, thereby promoting collaboration across different institutions and providing a practical tool to advance survival analysis in oncology research.

## INTRODUCTION

Time-varying covariates (TVCs) in survival analysis have gained interest in both statistical and medical literature studies.^1-13^ An example is the receipt of hematopoietic cell transplantation (HCT) after chimeric antigen receptor T-cell (CAR-T) infusion, which may occur at varying times or not at all.^5-8^ Standard Cox models and Kaplan-Meier (KM) curves, often called naïve methods, incorrectly assume that TVC status is known at baseline and may lead to biased results and potential conflicting conclusions. Landmark analysis offers an alternative but is limited by its dependence on a fixed landmark time. Time-dependent (TD) Cox is better suited for TVC, and a new visualization approach (Smith-Zee) constructs survival curves properly based on the TD Cox model.

**CONTEXT**

**Key Objective**
Are discrepancies in reported effects of post–chimeric antigen receptor T-cell hematopoietic cell transplantation and other time-varying covariates (TVCs) be explained by different statistical methods, and can a standardized tool enable reproducible TVC analysis across institutions?
**Knowledge Generated**
Time-dependent (TD) Cox is always preferred over naïve Cox for survival analyses with TVCs, even when results appear to be similar in practice. We developed TVCurve, R Shiny tool to compare naïve, landmark, and TD Cox models and generate survival curves. Its simulation panel illustrates biases from inappropriate methods. TVCurve breaks collaboration barriers since it does not require data sharing while ensuring standardized analyses across data sets.
**Relevance *(J.L. Warner)***
The authors have developed a survival analysis tool that should have a low barrier to use and implementation. Community input should be sought to quantify the usability and utility of the tool in the production setting.*
*Relevance section written by *JCO Clinical Cancer Informatics* Editor-in-Chief Jeremy L. Warner, MD, MS, FAMIA, FASCO.

Despite the existence of these methods, it is not uncommon to observe inappropriate use of the naïve Cox or KM curves in survival analysis with TVC as noted above.^8^ For instance, the role of HCT after CAR-T in patients with ALL is debatable.^6-8,14-18^ Previous analyses using TD Cox models or landmark analyses revealed that HCT is associated with improved leukemia-free survival (LFS).^6,7^ However, a retrospective study using the naïve KM method showed no association.^8^

We evaluate this from a unique angle: whether the discrepancies may be driven by the use of different statistical methods. Better understanding of reasons of these inconsistencies requires applying different models to the same data set. However, it faces challenges related to data sharing between different institutions and, more importantly, ensuring that consistent analytical codes are used across various data sets. To address these issues, we developed TVCurve, a one-of-a-kind, open-source R Shiny tool that enables appropriate models and survival curves for TVC analysis without coding expertise. Using TVCurve, we reanalyzed two CAR-T trials to assess whether statistical method choice explains previous inconsistencies.^6,7^ As an additional example, we applied TVCurve to evaluate the impact of chronic graft-versus-host disease (cGVHD) after transplant on relapse-free survival (RFS).

## METHODS

### Two CAR-T Studies NCT02028455 and NCT01865617

Our work was motivated by two CD19 CAR-T trials (Case Study [CS] 1 Seattle Children's: NCT02028455 and CS 2 Fred Hutch: NCT01865617).^6,7,17,18^ The primary end point, LFS, is defined as time from CAR-T to the first occurrence of relapse or death. Key variables, including the receipt of HCT after CAR-T, or response after CAR-T, occur after baseline and must be treated as TVCs.

We aimed to assess whether the conflicting conclusions about HCT's effect post–CAR-T arise from the chosen statistical methods or are intrinsically related to the individual study. Therefore, we applied multiple methods across both studies: naïve method, landmark analysis with two differing landmark times (median time of receiving HCT and 0.95 quantile time of receiving HCT), and TD Cox with the Smith-Zee curve.

### The Impact of cGVHD Presence (or absence) for Patients With Allotransplant (CS 3)

Furthermore, as another illustration example, we analyzed data from patients who underwent allotransplant at Fred Hutch between 2010 and 2015 (CS 3). The primary end point was RFS, defined as the time from transplant to the first occurrence of relapse or death, whichever occurred sooner (with censoring at the last follow-up for relapse-free survivors). The occurrence of cGVHD post-transplant was treated as a TVC. The effect of cGVHD on RFS after transplant has been previously explored.^19-21^ One study using landmark analysis with a 1-year landmark time (restricting analysis to patients who are relapse-free at 1 year after HCT) indicated a significant association between cGVHD and inferior RFS.^19^ By contrast, other studies,^20,21^ when using the TD Cox model, showed improved RFS associated with cGVHD or no discernible association. To better understand whether the different conclusions may be from the use of different methods, we applied the same set of methods as used in the CAR-T studies (naïve, landmark analysis, and TD Cox) to this cohort.

### Statistical Methods

The naïve Cox/KM model assumes that TVCs are known at baseline. For example, patients who receive HCT are immortal until the time of transplant, resulting in immoral time bias associated with the naïve method.^1-4^ Failing to account for the period between baseline and the TVC change can result in estimation bias, known as immortal bias.

Landmark analysis selects a fixed postbaseline time (eg, 3 months after infusion) as the landmark^9,10^ and classifies patients by their TVC status at the landmark time. However, this method ignores changes in TVC after the landmark and excludes patients who fail or are censored before the landmark, reducing power and introducing bias. Furthermore, the selection of a landmark time can influence results, creating an additional layer of variability.

The TD Cox model can robustly estimate TVC effects, but the visualization of survival curves remains challenging. Recently, methods such as the Smith-Zee^4^ and the Extended Kaplan-Meier (Extended KM)^22^ offer solutions. The Smith-Zee method creates hypothetical patients to derive survival curves from TD Cox models, maintaining statistical rigor.^4^ The Extended KM method, a nonparametric alternative, allows patients to switch groups as their TVC status changes.^5,22^

Note that for all methods involving the Cox model, proportional hazards assumption needs to be checked. The operation steps for each method are summarized in Table 1, and more details are included in the Data Supplement (Section A).

**TABLE 1.** Summary of Operational Steps of Statistical Methods for TVC Analysis

Model	Curve	Workflow	Recommendation
Naïve Cox	Naïve KM curve	1. Define *X*_fix_ = 1 if TVC status ever changes, 0 if no change 2. Fit Cox model/KM curve for the outcome by *X*_fix_	Not recommended
Landmark Cox	Landmark KM curve	1. Select a landmark time T* after day 0 and this T* becomes new time 0 in all the analyses 2. Define XT* = 1 if TVC changes by the landmark time T*; XT* = 0 if TVC does not change by the landmark time T*. XT* is fixed from the landmark time T* 3. Exclude patients who fail or censor before T* 4. Fit Cox model/KM curve for the outcome (with new starting time from T*) by XT*	Useful when outcome occurs late and TVC occurs early at a prespecified time
TD Cox	Smith-Zee curve	1. Reshape data into long format (counting process) with multiple time intervals per patient and corresponding TVC value at each time interval; use *tmerge* in *survival* R package 2. Fit the TD Cox model with *coxph* from *survival* R package on long-format data 3. Generate Smith-Zee plots based on the fitted model and mimic new patients whose TVC status changes at a prespecified time (*T*_change_) for visualization. Survival curves split at *T*_change_ to contrast patients who do and do not experience a TVC status change at this time *T*_change_	Preferred; provide unbiased estimation
TD Cox	Extended KM curve	1. Reshape data into long format, similar to above 2. Use the extended KM method, where patients' group is updated dynamically as determined by TVC status at a given time	Useful visualization complements to Smith-Zee

Abbreviations: KM, Kaplan-Meier; TD, time-dependent; TVC, time-varying covariate.

### R Shiny Software TVCurve

TVCurve is an open-source R Shiny tool for survival analysis with TVCs, incorporating multiple models (naïve Cox, landmark Cox, and TD Cox) and curves (naïve KM, landmark KM, Smith-Zee, and Extended KM) to provide a comprehensive suite for TVC analysis.

Although landmark analysis and the TD Cox method are established methods for analyzing TVCs, their practical application, especially visualization of the survival curves by TVCs, may not always be straightforward. The biases and limitations of different methods may not be well-understood, particularly among users without coding experience. TVCurve addresses these challenges with user-friendly features.

1. Intuitive interface: Users can upload data, define end points, and choose a model for TVC analysis. With a strong emphasis on data privacy, it ensures that results are exclusively presented to the user who provided the data. Users cannot access the server's file system or underlying code, temporary files are deleted after loading, and no data are logged or retained beyond that session.
2. Comprehensive methods and curves: TVCurve incorporates a range of methods and curves and provides a model summary for each method. Source codes are available upon request. A comparison panel highlights the differences between the robust methods (TD Cox and Smith-Zee) and the less appropriate methods (naïve Cox and naïve KM).
3. Simulation panel: Users can simulate data by adjusting parameters, such as sample size, shape and rate parameters for the Weibull distribution of time to outcome event (*α*_E_, *λ*_E_) and time to TVC (*α*_TVC_, *λ*_TVC_), and the log hazard ratio (HR) for the TVC effect (*β*_TVC_). The Weibull distribution, favored for modeling survival rates in oncology research,^3,11^ was used in alignment with the simulation setting from the study by Mi et al.^3^ As a novel feature, we provide contour plots for illustrating HR bias under various scenarios (Data Supplement, Section C and Fig S5).
4. TVCurve is available online at Shiny Applications Online,^23^ with a video that provides a step-by-step user guide.^24^

## RESULTS

### Using Appropriate Statistical Models Is Important Although Different Models Yield Similar Results for the Effect of HCT After CAR-T Infusion

**FIG 1.**
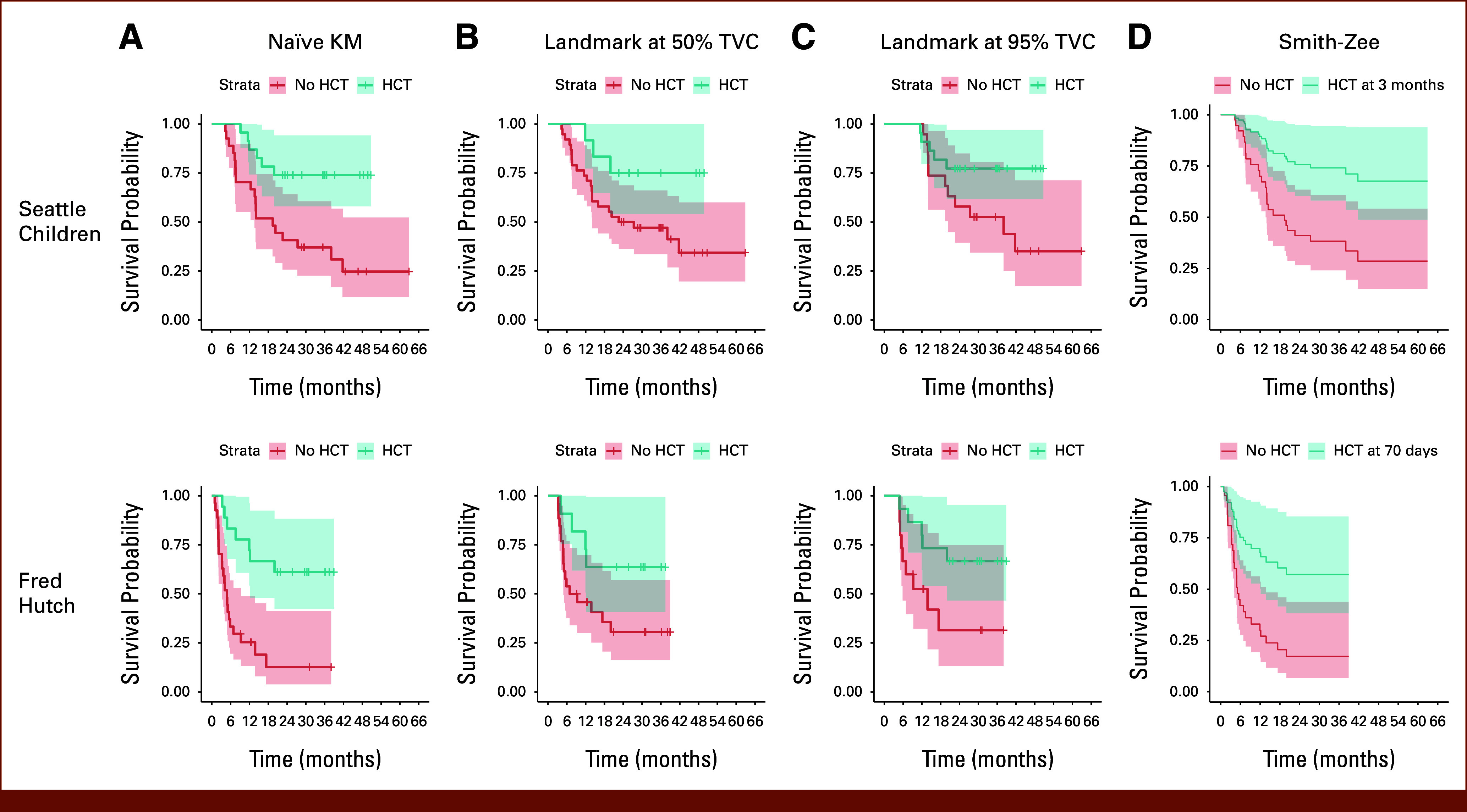
Using different survival models and visualization tools to analyze the association between HCT after CAR-T infusion and LFS on Seattle Children's PLAT-02 data (CS 1) with n = 50 (top) and Fred Hutch Protocol 2639 data (CS 2) with n = 45 (bottom). (A) Naïve KM curve (not recommended). (B) Landmark KM curve with landmark time at the median time of receiving HCT in each data set (50% TVC; tentative). (C) Landmark KM curve with landmark time at 0.95 quantile time of receiving HCT in each data set (95% TVC; tentative). (D) Smith-Zee plot based on the TD Cox model (recommended). CAR-T, chimeric antigen receptor T cell; HCT, hematopoietic cell transplantation; KM, Kaplan-Meier; LFS, leukemia-free survival; TD, time-dependent; TVC, time-varying covariate.

In CS 1,^7,17^ among 50 pediatric and young adult patients who achieved CR after CAR-T infusion, 46% underwent HCT postinfusion. Results from different statistical methods are summarized in Figure 1 and Table 2. Findings from the naïve KM and naïve Cox models, although deemed unsuitable for analyzing the TVC, aligned with the TD Cox model (naïve Cox HR = 0.28/*P* < .01 and TD Cox HR = 0.31/*P* = .01). Landmark analysis revealed distinct results based on the different landmark times (3 months *v* 9 months after CAR-T infusion), demonstrating limitations of this method (HR = 0.34/*P* = .07 for the landmark at 3 months *v* HR = 0.57/*P* = .26 for the landmark at 9 months). In CS 26,^6,18^ with 45 adult patients with ALL (18 receiving HCT post–CAR-T), we observed similar HRs and *P* values across all the models. Table 2 shows that HR = 0.23/*P* < .01 in the naïve Cox model and HR = 0.31/*P* = .01 in the TD Cox model and HR = 0.38/*P* = .07 for the landmark time at 70 days (median time of receiving HCT) and HR = 0.35/*P* = .06 for the landmark time at 135 days (0.95 quantile time of receiving HCT). The consistent results obtained from naïve and TD Cox models in both CAR-T studies suggest that variations in the conclusion of the impact of the HCT effect across studies may not be due to the analytical methods used but could be inherent to the studies themselves.^6-8^ While the consistency in results across methods may be reassuring, we recommended the TD Cox model for analyzing the effect of HCT after CAR-T infusion; landmark analysis may be useful in other scenarios, such as evaluating the effect of response at a predefined time point (eg, day 28 after CAR-T) that is soon after baseline and much earlier than possible event times.^5^

**TABLE 2.** Different Cox Models With TVC Have Been Conducted on Three CSs

Model	HR	95% CI	*P*
CS 1: Seattle Children's PLAT-02 (n = 50)			
Naïve Cox	0.28	0.11 to 0.71	<.01
Landmark Cox			
Landmark time 3 months	0.34	0.10 to 1.14	.07
Landmark time 9 months	0.57	0.21 to 1.54	.26
TD Cox	0.31	0.12 to 0.79	.01
CS 2: Fred Hutch protocol 2639 (n = 45)			
Naïve Cox	0.23	0.10 to 0.56	<.01
Landmark Cox			
Landmark time 70 days	0.38	0.13 to 1.14	.07
Landmark time 135 days	0.35	0.12 to 1.08	.06
TD Cox	0.31	0.12 to 0.78	.01
CS 3: Patients after transplant (n = 1,528)			
Naïve Cox	0.45	0.39 to 0.52	<.0001
Landmark Cox			
Landmark time 6 months	1.08	0.89 to 1.31	.44
Landmark time 17 months	1.25	0.95 to 1.63	.11
TD Cox	0.95	0.80 to 1.13	.57

NOTE. CS 1: Seattle Children's PLAT-02 study with pediatric patients with ALL and TVC is receiving HCT after CAR-T infusion; CS 2: Fred Hutch Protocol 2639 adult patients with ALL and TVC is receiving HCT after CAR-T infusion; CS 3: Fred Hutch study for patients who received allotransplant between 2010 and 2015 and TVC is cGVHD occurrence after HCT. The models include naïve Cox regression, landmark analysis with the Cox model, and the TD Cox model. The landmark analysis used two versions of landmark times: (1) Median time of TVC status change in each data set, that is, 3 months for CS 1 data, 70 days for CS 2 data, and 6 months for CS 3 data; (2) 0.95 quantile time of TVC status change in each data set, that is, 9 months for CS 1 data, 135 days for CS 2 data, and 17 months for CS 3 data. HR, CI, and *P* values are reported.

Abbreviations: CAR-T, chimeric antigen receptor T cell; cGVHD, chronic graft-versus-host disease; CS, Case Study; HCT, hematopoietic cell transplantation; HR, hazard ratio; TD, time-dependent; TVC, time-varying covariate.

### Statistical Models Vary in Their Conclusions About the Impact of cGVHD Presence (or Absence) for Patients With Allotransplant

**FIG A1.**
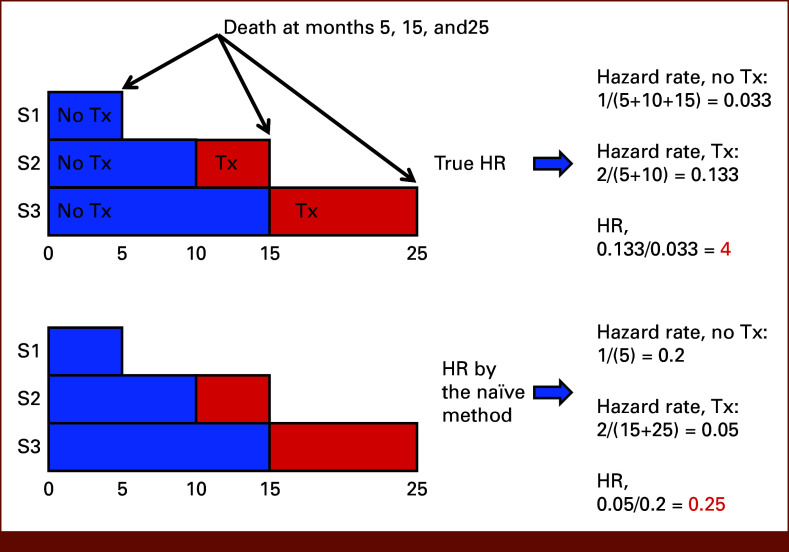
Example to show the biased HR estimate from the naïve method against the true value. Assume there are three patients S1, S2, and S3 who died at months 5, 15, and 25, respectively. S1 did not receive any treatment (Tx), S2 received Tx at the 10th month, and S3 received Tx at the 15th month. The upper plot shows the true Tx status change (which should be a TVC), where blue block is the correct “no Tx” period that should be used to estimate the true HR. The lower plot shows how the HR is estimated in the naïve method, which incorrectly considers all the time periods from S2 and S3 as Tx period. TVC, time-varying covariate.

For the two CAR-T CSs, the effect of HCT appears to be consistent across various methods. However, such consistency is not universally observed. As illustrated in Appendix Fig A1, if we mistakenly treated patients' TVC status change as they were known at baseline, the HR estimate from the naïve method can be in the opposite direction of the true value. CS 3 is presented as another illustration example. We examined the impact of cGVHD after transplant on RFS of this cohort of n = 1,528 patients (51% of whom developed cGHVD) using TVCurve.

**FIG 2.**
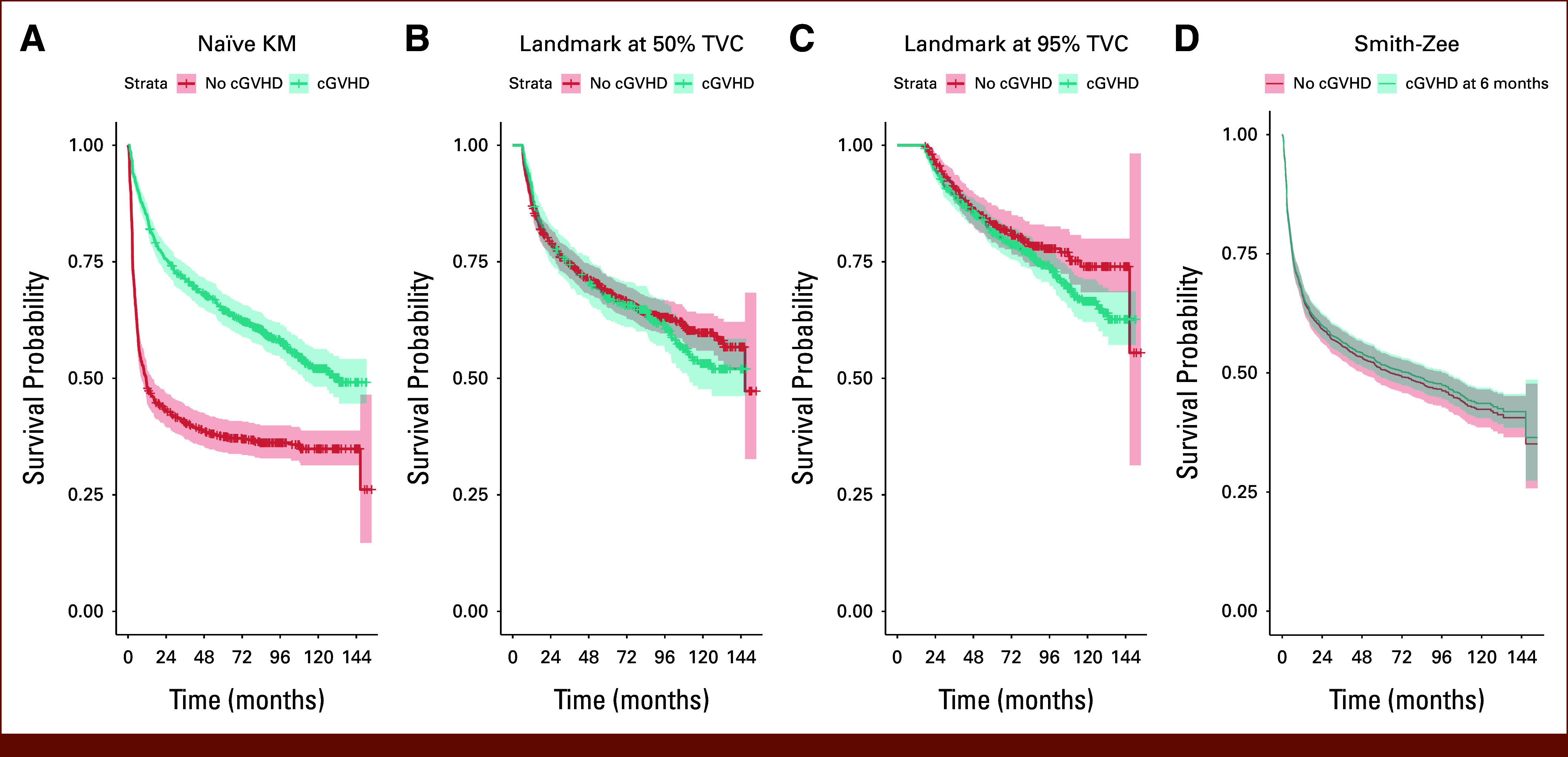
Using different survival models and visualization tools to analyze the association between cGVHD after HCT and RFS on n = 1,528 patients who got allotransplant at Fred Hutch between 2010 and 2015 (CS 3). (A) Naïve KM curve (not recommended). (B) Landmark KM curve with landmark time at the median time of cGVHD occurrence (50% TVC; tentative). (C) Landmark KM curve with landmark time at 0.95 quantile time of cGVHD occurrence (95% TVC; tentative). (D) Smith-Zee plot based on the TD Cox model (recommended). cGVHD, chronic graft-versus-host disease; CS, Case Study; HCT, hematopoietic cell transplantation; KM, Kaplan-Meier; RFS, relapse-free survival; TD, time-dependent; TVC, time-varying covariate.

Not surprisingly, the naïve method showed that cGVHD is significantly associated with improved RFS with HR = 0.45/*P* value <.0001 (Fig 2; Table 2). Conversely, both the TD Cox model and the Smith-Zee curves indicated no association between cGVHD and RFS, as shown in Table 2 (HR = 0.95/*P* = .57). For landmark analysis, the results were slightly different based on the landmark time selected, yet neither presented a significant cGVHD effect on RFS (HR = 1.08/*P* = .44 with a landmark at 6 months, the median time of cGVHD occurrence, and HR = 1.25/*P* = .11 with a landmark at 17 months, 0.95 quantile time of cGVHD occurrence). This example shows how inappropriate use of statistical methods that do not account for immortal time bias can lead to incorrect conclusions. This example also shows how results from a landmark analysis can differ based on the choice of landmark times.

### Naïve Method Yielded Greater Bias When TVC Occurs Late and Outcome Events Occur Early

**FIG 3.**
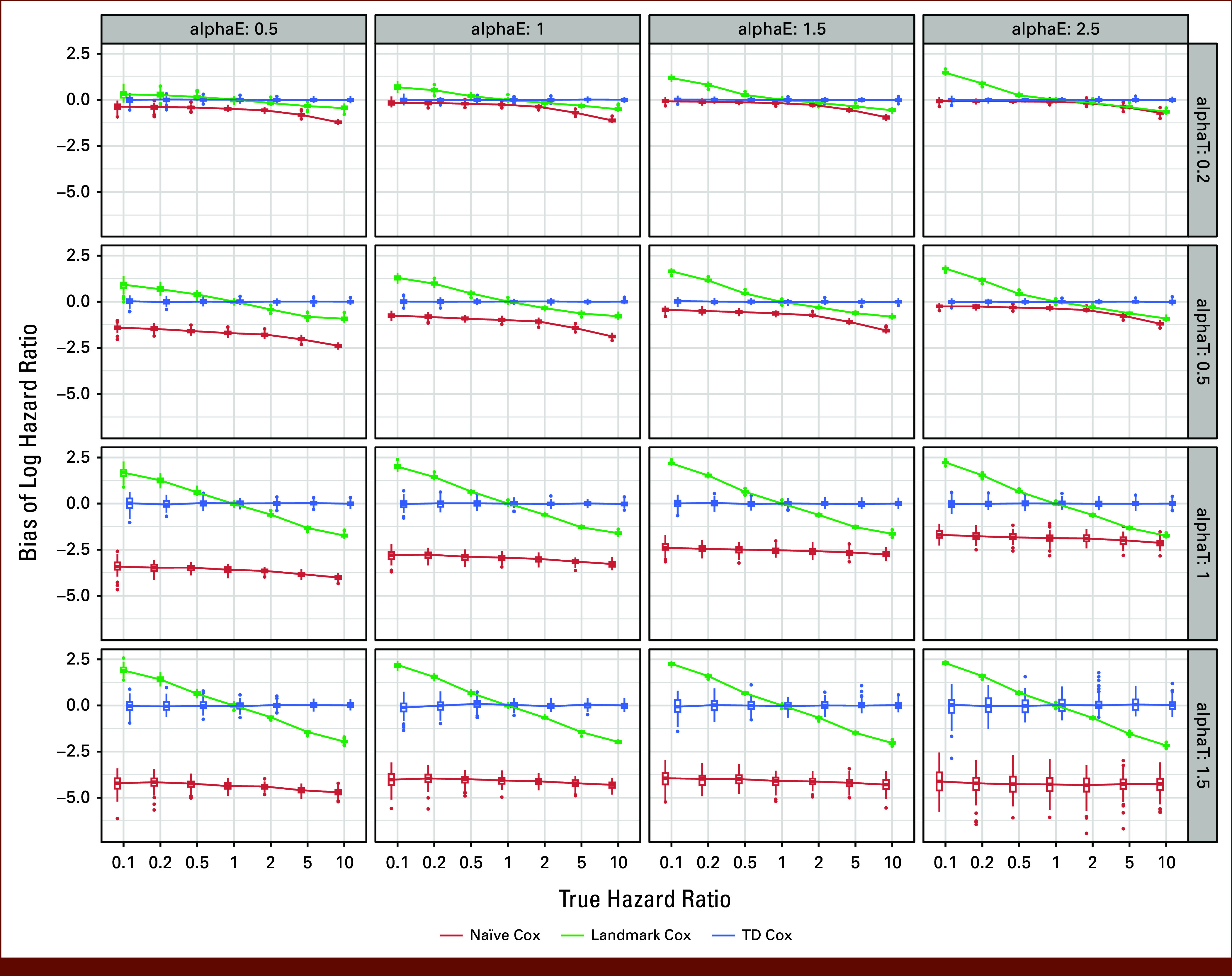
Bias of the estimated log HR, by different combinations of simulation parameter (various alphaE, alphaT, true hazard ratio values), across the naïve method, landmark analysis, and TD Cox model (lambdaT = 0.05, lambdaE = 0.005, number of simulations = 100, sample size n = 1,000). HR, hazard ratio; TD, time-dependent.

**FIG A2.**
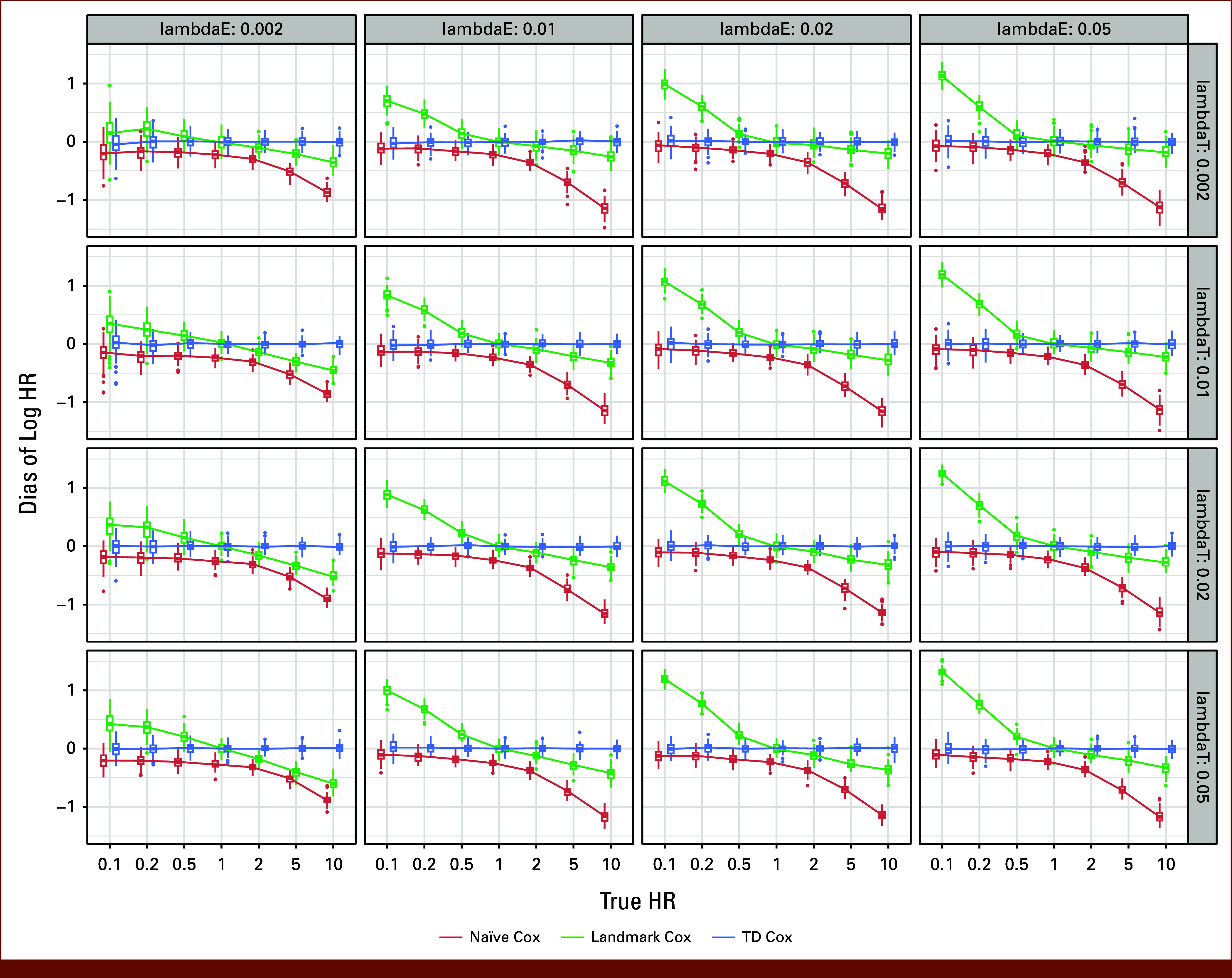
Bias of the estimated log HR, by different combinations of simulation parameter, such as various lambdaE (*λ*_E_), lambdaT (*λ*_TVC_), and true HR ($\beta_{\text{true}}$) values across the naïve method, landmark analysis, and TD Cox model (alphaE *α*_E_ = 1, alphaT *α*_TVC_ = 0.2, number of simulations = 100, N = 1,000). HR, hazard ratio; TD, time-dependent; TVC, time-varying covariate.

**FIG A3.**
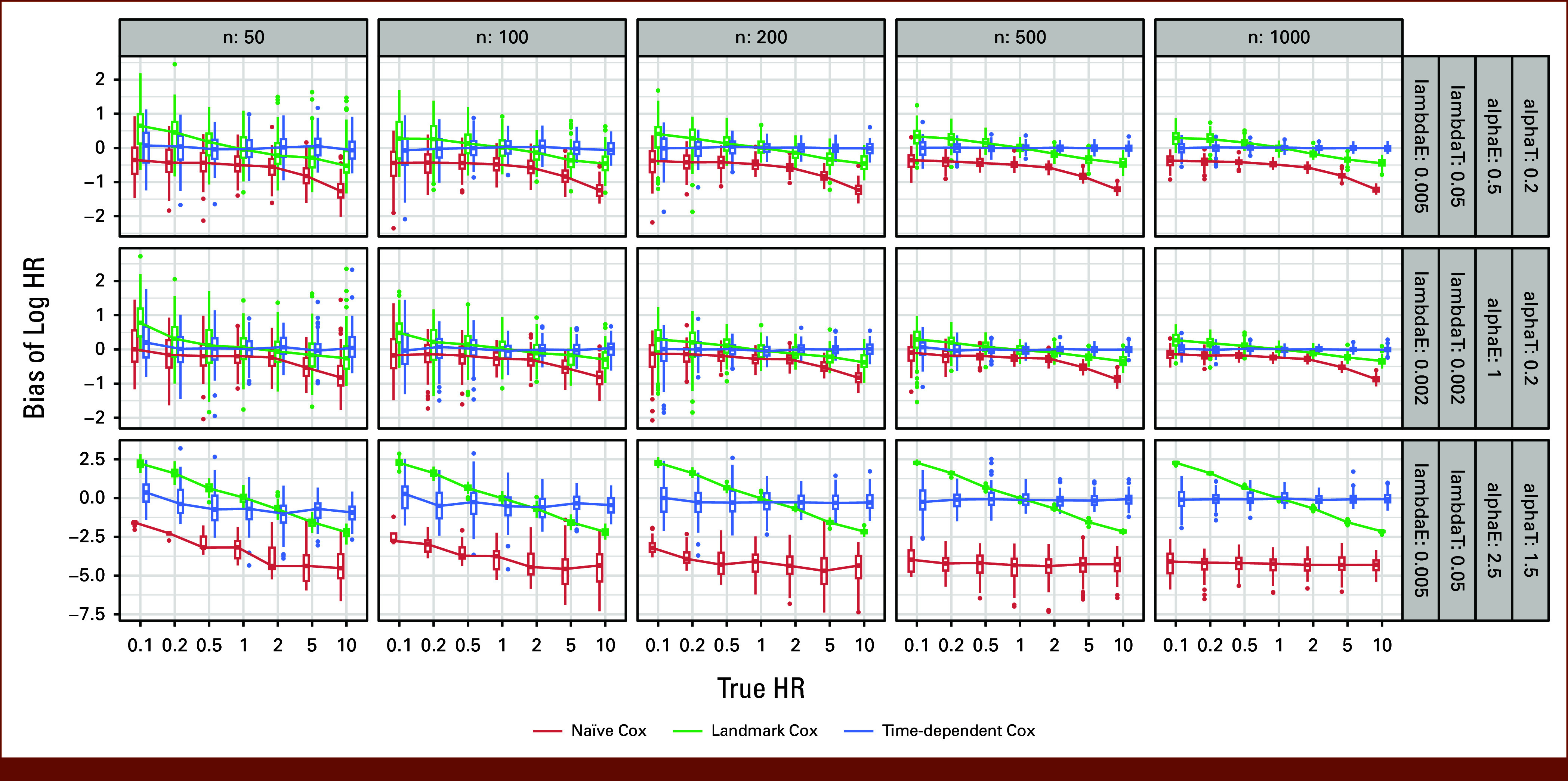
Bias of the estimated log HR, by different combinations of simulation parameter, such as various sample size (N), lambdaE (*λ*_E_), lambdaT (*λ*_TVC_), alphaE (*α*_E_), alphaT (*α*_TVC_), and true HR ($\beta_{\text{true}}$) values, across the naïve method, landmark Analysis, and TD Cox model (number of simulations = 100). HR, hazard ratio; TD, time-dependent; TVC, time-varying covariate.

**FIG A4.**
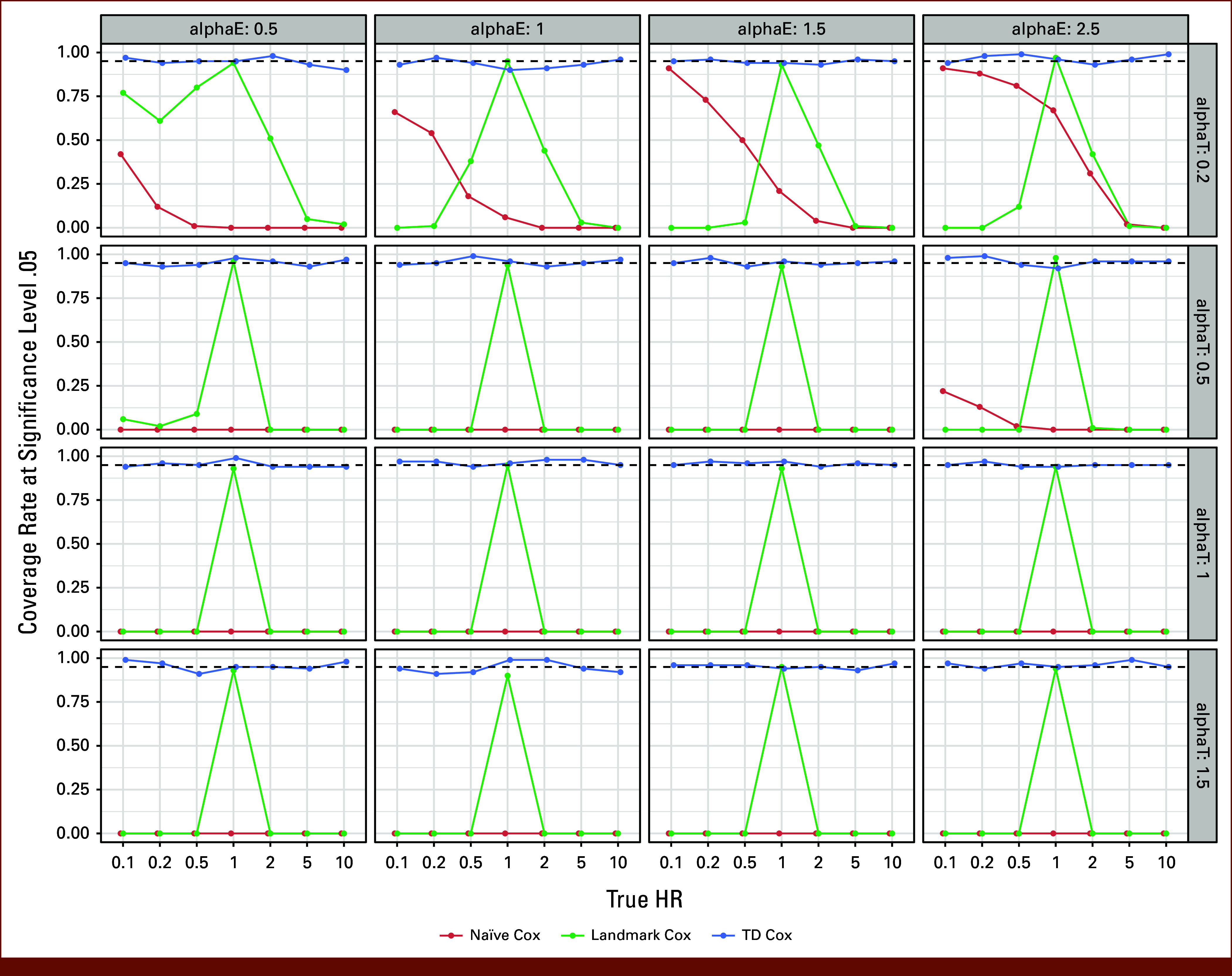
Coverage rate of 95% CI for log HR, by different combinations of simulation parameter (various alphaE, alphaT, true HR values), across the naïve method, landmark analysis, and TD Cox model. HR, hazard ratio; TD, time-dependent; TVC, time-varying covariate.

We conducted rigorous simulations to compare various statistical methods as detailed in the Data Supplement (Section B). Figure 3 shows the bias of log HR (ie, $\hat{\beta }-\beta_{\text{true}}$), and Appendix Fig A4 shows the coverage rate of the 95% CI of the estimated HR. Figure 3 and Appendix Fig A4 show pronounced bias from naïve methods in most of the scenarios. Notably, log HR $\hat{\beta }$ from the naïve Cox model persistently underestimated the true log HR ($\hat{\beta }-\beta_{\text{true}}<0)$, which is expected since the naïve method inappropriately assumes that TVC status is known at baseline. This misclassifies the period from baseline to TVC status change as exposed, while it is actually unexposed, ie, such patients are immortal until the receipt of HCT, resulting in immortal time bias. Consequently, event rates appear lower in the exposed group and higher in the unexposed, causing a smaller HR estimate than the true value. This conclusion aligns with that in the study by Mi et al,^3^ which compared the naïve method, landmark analysis, and the Mantel-Byar method (a special case of TD Cox). The magnitude of bias varies by $\beta_{\text{true}}$ and is sensitive to the shape parameters (*α*_E,_ *α*_TVC_) but showed little dependence on the rate parameter *λ*_E_ or *λ*_TVC_ or sample size N (Appendix Figs A2 and A3).

### The Bias in the Naïve Method and Landmark Analysis Was Minimal When the Majority of Outcome Events Occur Late and TVC Status Changes Early

If TVC changed quickly (smaller *α*_TVC_) and most outcome events occurred late (larger *α*_E_), immortal time bias by the naïve method is minimal. This explains the less evident bias observed in two CAR-T studies where the TVC (post–CAR-T infusion HCT) occurred earlier (the median time of receiving HCT is 2.3 months and 0.95 quantile is 4.4 months in CS 2; the median time of receiving HCT is 3 months and 0.95 quantile is 9 months in CS 1) and most events occurred relatively late (patients achieving MRD-CR typically experience extended LFS). However, in the cGVHD study, noticeable bias was seen with the naïve method because the TVC cGHVD occurred much later (median 6 months and 0.95 quantile 17 months), while RFS events could occur early.

**FIG A6.**
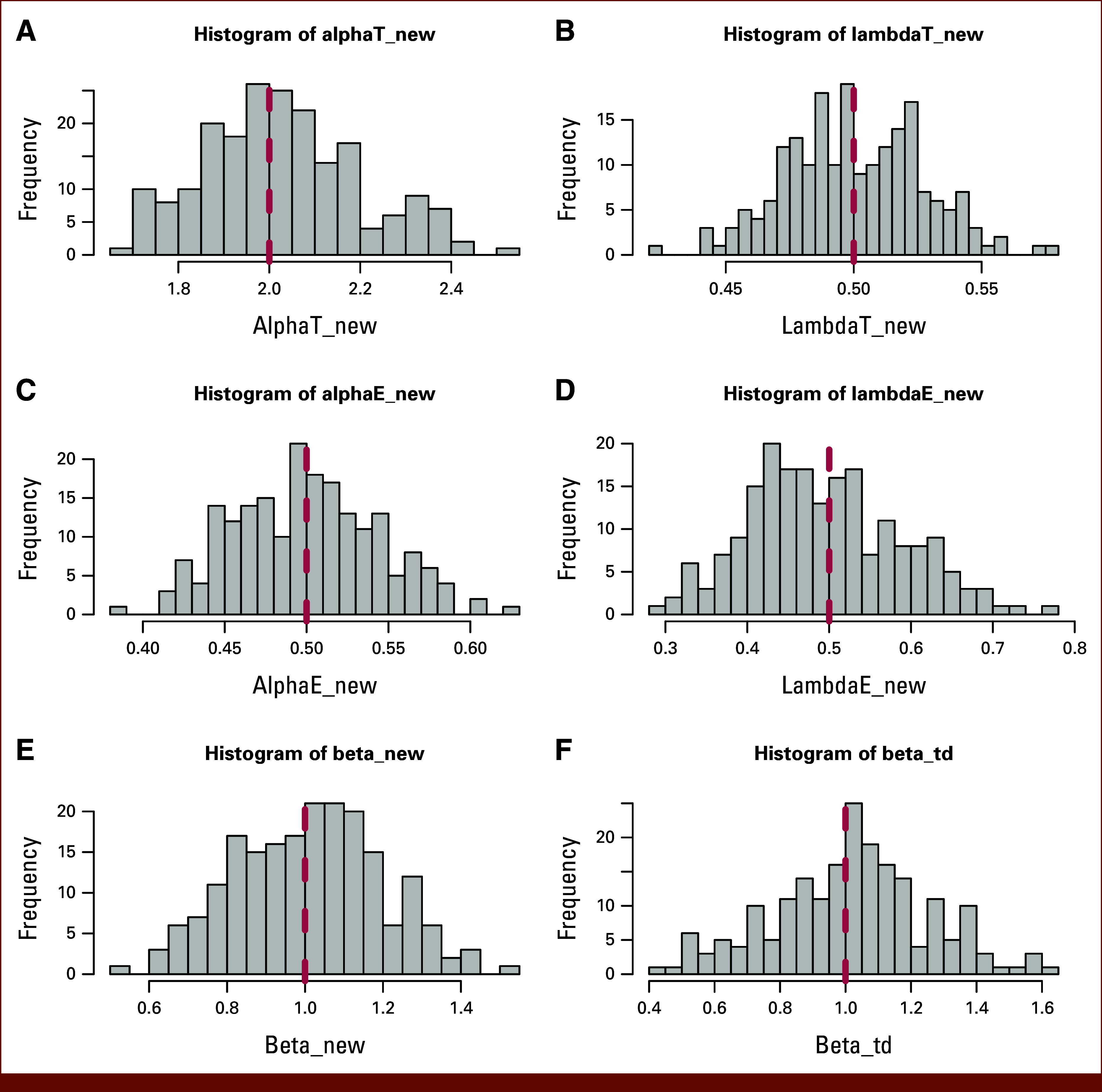
Simulation result for the new Weibull model fitted using the maximum likelihood approach, and TD Cox model using 100 simulations. (A)-(E) Histogram of the estimated Weibull parameters ${\hat{a}}_{\text{TVC}}$, ${\hat{l}}_{\text{TVC}}$_,_ ${\hat{a}}_{E}$, ${\hat{l}}_{E}$, and log HR $\hat{\beta }$ from the maximum likelihood approach. (F) Histogram of the estimated log HR ${\hat{\beta }}_{\text{TDCox}}$ from the TD Cox model. The vertical red line indicates the true value of the parameters. HR, hazard ratio; TD, time-dependent; TVC, time-varying covariate.

For landmark analysis, when outcome events occur late and much later than TVC (eg, when *λ*_E_ is small, see Appendix Fig A2) or true HR ≈ 1 (Fig 3), bias is smaller. This explains the less bias observed in CS 2 by landmark analysis, where 95% of TVC occurred relatively early (4.4 months) and most events came later than TVC. The choice of landmark times influences magnitude of bias: in CS 1, bias increased with a late landmark (0.95 quantile of 9 months) because TVC (HCT) occurred later for some patients, so outcome events appear to be relatively early. Therefore, when TVC change occurs shortly after baseline and there is a predefined early assessment for all patients, landmark analysis is most useful. For instance, in CAR-T trials for patients with ALL, response should be considered as a TVC and is typically evaluated on day 28 and death or censoring before day 28 is rare, making day 28 a reasonable landmark time.^5^ In simulations, bias from the naïve method is subtle when *α*_E_ is large and *α*_TVC_ is small. However, in real-world application, this information is rarely available. Thus, we developed a novel algorithm that can precisely estimate the parameters (${\hat{\alpha }}_{E},{\hat{\lambda }}_{E},{\hat{\alpha }}_{\text{TVC}},{\hat{\lambda }}_{\text{TVC}}$) and log HR ${\hat{\beta }}_{\text{true}}$ directly from real data (not simulated data) via a maximum likelihood approach. The full workflow is given in the Data Supplement (Section D), with simulation results in Appendix Fig A6.

### Summary of Bias Patterns for Each Method

Table 3 summarizes these patterns based on the simulation results. In general, the TD Cox model yields unbiased estimates, and the naïve method consistently underestimates the hazard for the exposed group, whereas bias is minimal when more TVC exposure occurs sooner and more outcomes occur later. For the landmark method, the direction of bias varies and bias is minimal when there is truly no association between TVC and outcome, and landmark can be appropriate if all events occur late and TVC occurs early.^5^

1. When the true HR = 1 ($\beta_{\text{true}}=0$),
  - Naïve method: Estimates fall below 0 ($\hat{\beta }$ << 0), incorrectly suggesting that exposure is protective.
  - Landmark method: Estimates remain close to 0 ($\hat{\beta }$ ≈ 0) with minimal bias.
2. When the true HR > 1 ($\beta_{\text{true}}>0$),
  - Naïve method: Estimates are biased toward the null ($\hat{\beta }\approx 0$) and incorrectly suggest no association, while exposure actually increases hazard
  - Landmark method: Estimates $\hat{\beta }$ are lower than $\beta_{\text{true}}$ (or even opposite direction) and incorrectly suggest no association or protective, while exposure actually increases hazard
3. When the true HR < 1 ($\beta_{\text{true}}<0$),
  - Naïve method: Estimates exaggerate the protective effect ($\hat{\beta }$ << 0) and incorrectly suggest a stronger protective effect, while it is actually weaker
  - Landmark method: Estimates $\hat{\beta }$ are higher than $\beta_{\text{true}}$ (or may suggest increased risk), underestimating the true protective effect.
4. When TVC occurs early (small *α*_TVC_) and events occur late (large *α*_E_), such as *α*_TVC_ ≤ 0.5 and *α*_E_ ≥ 1.5 in Figure 3
  - Naïve method: $\hat{\beta }$ ≈ $\beta_{\text{true}}$, minimal bias
  - Landmark method: the direction of bias varies, and bias is minimal when $\beta_{\text{true}}$ ≈ 0
5. When TVC occurs late (large *α*_TVC_) or events occur early (small *α*_E_), such as *α*_TVC_ ≥1 or *α*_E_ ≤1 in Figure 3
  - Naïve method: Large bias, estimates that $\hat{\beta }$ is much lower than $\beta_{\text{true}}$ and always underestimates the hazard for the exposed group
  - Landmark method: Large bias, $\hat{\beta }-\beta_{\text{true}}$ >0 when $\beta_{\text{true}}<0;\;\hat{\beta }-\beta_{\text{true}}$ <0 when $\beta_{\text{true}}>0$, and $\hat{\beta }$ ≈ $\beta_{\text{true}}$ when $\beta_{\text{true}}$ ≈ 0.

**TABLE 3.** Summary of Direction of Bias and Implication for the Conclusion for Various Methods, Based on Simulation Results

Scenario	TD Cox	Naïve	Landmark
Summary	Unbiased	Always bias toward lower hazard for the exposed group; bias is minimal when TVC exposure occurs sooner, and outcome occurs later	The bias direction varies; bias is minimal when there is truly no association between TVC exposure and outcome
More TVC occurs early (small *α*_TVC_), more events occur late (large *α*_E_)	Unbiased	Minimal bias	The bias direction varies; bias is minimal when $\beta_{\text{true}}\approx 0$
More TVC occurs late (large *α*_TVC_), more events occur early (small *α*_E_)	Unbiased	Large bias; underestimates hazard for the exposed group	The bias direction varies; bias is minimal when $\beta_{\text{true}}\approx 0$
When $\beta_{\text{true}}$ = 0 (HR = 1)	Unbiased	Estimated $\hat{\beta }$ <0 (HR < 1); bias away from null; could incorrectly suggest that exposure is protective, while there is actually no association	Estimated $\hat{\beta }$ close to 0 (HR close to 1); minimal bias
When $\beta_{\text{true}}$ >0 (HR > 1)	Unbiased	Estimated $\hat{\beta }$ closer to 0 (HR closer to 1); bias toward null; could incorrectly suggest no association while exposure actually increases hazard	Estimated $\hat{\beta }$ smaller than $\beta_{\text{true}}$; bias toward null (HR closer to 1) or to an opposite direction (HR < 1); could incorrectly suggest no association or protective, while exposure actually increases hazard
When $\beta_{\text{true}}$ <0 (HR < 1)	Unbiased	Estimated $\hat{\beta }$ further from 0 (HR farther from 1); bias away from null; could incorrectly suggest a stronger protective exposure effect while the effect is actually weaker	Estimated $\hat{\beta }$ larger than $\beta_{\text{true}}$; bias toward null (HR closer to 1) or to an opposite direction (HR > 1); could incorrectly suggest no association or a higher outcome risk associated with exposure, while exposure is actually protective

Abbreviations: HR, hazard ratio; TD, time-dependent; TVC, time-varying covariate.

This summary provides a quick reference for understanding the bias direction and its clinical implications.

## DISCUSSION

Clinical research relies on robust computational tools that allow investigators to conduct data analyses and precisely report findings. TVCurve stands out by offering a comprehensive suite of models, curves, and source codes for TVC analysis. Its drag-and-drop interface makes it approachable for users with limited coding experience, whereas R-experts can easily copy and paste source codes in R to customize analyses and simulations. Unlike other simulations with only static, snapshot-like results, TVCurve provides a dynamic simulation panel, allowing users to adjust parameters and immediately observe updated results.

Our results demonstrate that TVCurve facilitates accurate survival modeling (TD Cox) with TVCs, particularly showing scenarios in which Naïve and landmark methods can yield biased results. We also emphasize that the TD Cox model is always preferred over the naïve Cox model, even if the conclusions from the two methods are not qualitatively different. Landmark analysis is most useful when TVC status is evaluated soon after baseline for all patients (eg, day 28 CAR-T response).

In response to user's needs for multivariable analysis, we have expanded TVCurve beyond its original framework which is limited to univariable models with a single external binary TVC. It includes examples of multivariable TD Cox models with multiple TVCs or baseline covariates and step-by-step R code to guide users. We acknowledge that more complex cases where covariates change multiple times remain beyond the scope of this study and need future development. While user-friendly, a basic understanding of survival analysis is essential. We recommend involving a biostatistician in the study to ensure correct interpretation. We have used TVCurve for TVC analysis in published and ongoing research^7,25^ and present TVCurve to clinical and statistical audience at the Tandem meeting^26^ and ENAR conference,^27^ where we received positive comments and strong interest in broader applications. These early experiences underscore the usability and relevance of TVCurve across both biostatistical and clinical communities.

In conclusion, TVCurve fills a critical gap by providing an open-source, standardized tool for TVC analysis, advancing cancer research and clinical decision making. We recommend the TD Cox model and Smith-Zee curves for modeling TVCs such as HCT post–CAR-T, treatment response, B-cell recovery, and cGVHD after transplant. Importantly, TVCurve supports standardized analysis without requiring data transfer, promoting collaboration across institutions.

## Figures and Tables

**FIG A5.**
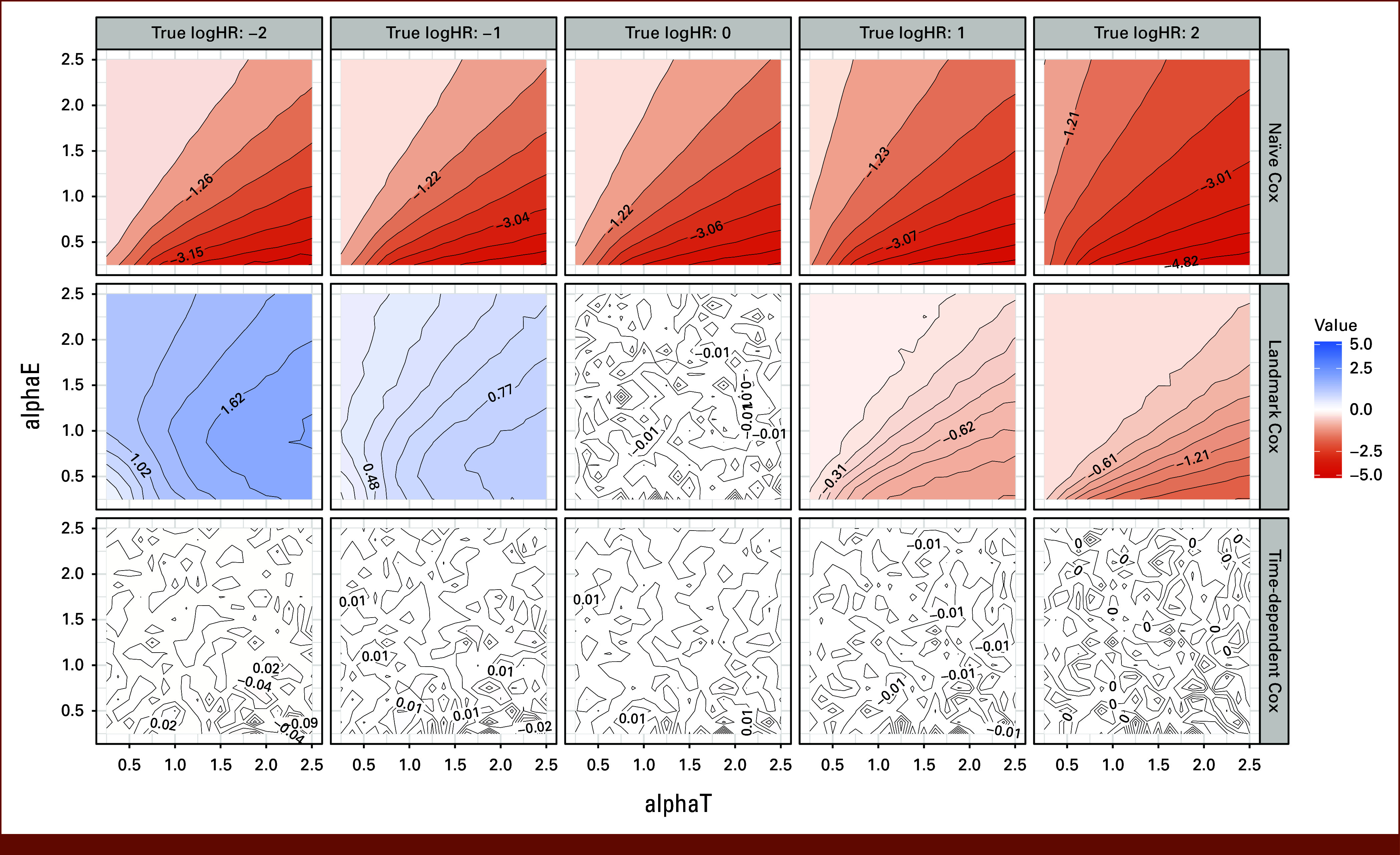
Bias contour based on different combinations of the parameters alphaE and alphaT, while fixing lambdaT and lambdaE at 0.025 and true log HR at different values (–2, –1, 0, 1, 2), across the naïve method, landmark analysis, and TD Cox model. HR, hazard ratio; TD, time-dependent.

## APPENDIX

### Workflow of Each Statistical Model

In this section, we briefly introduce the workflow and key idea for each survival model and how they conducted TVC analyses and the corresponding visualization in TVCurve. We included operational Table 1 in the main File.

1. Naïve Cox model and naïve KM curve
  This method is not recommended to analyze TVC association with time-to-event end points in survival analysis. But it is important to understand the bias in this approach that is caused by assuming that future TVC information is known at baseline.
  - Step 1: Consider TVC (eg, postinfusion HCT) as a fixed known binary covariate (*X*_fix_, Yes *v* No, where *X*_fix_ is coded as Yes if TVC ever occurred during the follow-up)
  - Step 2: Build a naïve Cox model between *X*_fix_ and the time-to-event end point.
  - Step 3: Generate a naïve KM curve to show the survival difference by *X*_fix_.
2. Landmark analysis with the Cox model and the KM curve
  - Step 1: Choose a fixed time *T** after day 0 (eg, 3 months after CAR-T infusion) as the landmark time.
  - Step 2: Create a fixed binary covariate, *XT**, which is defined according to patient's TVC status at landmark time *T** and it will not change. For instance, if a patient received HCT at 6 months, but the landmark *T** is chosen at 3 months, this patient's *XT** will be defined as “No HCT” because at 3 months, this patient did not receive HCT.
  - Step 3: Exclude patients who die or are censored before the landmark time.
  - Step 4: The time-to-event end point is updated to start at *T**. Build a standard Cox model and KM curves to show the survival difference by *XT** since *XT** is known at the new starting time (landmark time *T**).
3. TD Cox model and Smith-Zee curve
  - Step 1: We will reshape the data to allow each patient to have multiple rows to indicate a time interval with start and end and show the possible TVC status changes across time intervals, which is known as the counting process (long) format. The tmerge function from survival R package can handle this data transformation.
  - Step 2: Generate a TD Cox model using the long data from step 1. The coxph function from survival package can run the TD Cox model and provide HR, CI, and *P* value on the TVC.
  - Step 3: Create Smith-Zee plots based on the TD Cox model and mimic new patients whose TVC status changes at a prespecified time (*T*_change_) for visualization. The survival curves will split at the prespecified time *T*_change_ to compare survival probability between patients who do and do not experience a TVC status change at this time *T*_change_. The user can generate two curves by choosing one time (eg, postinfusion HCT at 3 months *v* no HCT) or multiple curves by choosing multiple times (eg, no HCT *v* HCT at 2 months *v* HCT at 4 months *v* HCT at 6 months).^4,7^
4. TD Cox model and extended KM curve
  - Step 1: Similar to Method 3, the data need to be reshaped to the long format.
  - Step 2: Use the extended KM approach^14^ to calculate the KM estimates, where patients' group affiliation is determined based on TVC status at a given time and is allowed to change.

### Simulation Panel on TVCurve

The above examples show that inappropriate methods for modeling TVC can lead to conclusions that are qualitatively the same as appropriate methods or conclusions that are qualitatively different. Even in the case where conclusions are qualitatively the same, we emphasize that the appropriate methods should be used. However, to explore what situations would lead to qualitatively similar or disparate conclusions, we performed simulation studies through the Simulation panel on TVCurve. We assume that the time-to-event end point follows a Weibull distribution characterized by shape parameter *α*_E_ and rate parameter *λ*_E_. The time to TVC status change also follows a Weibull distribution, but with distinct parameter *α*_TVC_ and *λ*_TVC_. The Weibull distribution, favored for modeling survival rates in oncology research,^3,11^ was used in alignment with the simulation setting from the study by Mi et al.^3^ Here, *α*_TVC_ represents whether the TVC status change is likely to occur at an earlier time or a later time, for example, *α*_TVC_ < 1 indicates that the hazard rate of TVC status changes is higher at an earlier time, *α*_TVC_ = 1 indicates that the hazard rate of TVC status change is constant over time (when *α* = 1, Weibull distribution becomes the exponential distribution) and *α*_TVC_ > 1 indicates that the hazard rate of TVC status change is higher at a later time. Similarly, the shape parameter *α*_E_ represents the shape of the hazard function for the outcome, with *α*_E_ > 1 indicating hazard of outcome increasing over time (ie, the rate of event occurrence is higher at later times) and *α*_E_ < 1 indicating hazard of outcome decreasing over time (ie, the rate of event occurrence is higher at an earlier time).^3^

### Using Contour Plots to Expose Variability in Bias of the Naïve Method Under Diverse Simulation Conditions

Contour plots show how the estimated log HR bias ($\hat{\beta }-\beta_{\text{true}}$) varies with combinations of two parameters, specifically, *α*_TVC_ and *α*_E_. Without loss of generality, given that *λ*_E_ = *λ*_TVC_ = 0.025 and *α*_TVC_ and *α*_E_ change from 0 to 2.5 in 0.1 increments with different log HR $\beta_{\text{true}}$ (–2, –1, 0, 1, 2), the following observations are made in Appendix Fig A5:

1. Naïve Cox: minimal bias is observed when *α*_E_ is high and *α*_TVC_ is low. Notably, for negative $\beta_{\text{true}}$ (eg, $\beta_{\text{true}}=-2$), a broad region shows minimal bias (ie, lightest orange color triangle region in Appendix Fig A5, the top-left panel: beta = –2, the region for *α*_E_ > 0.25 and *α*_TVC_ < 1.5). However, for $\beta_{\text{true}}=1$, this region shrinks significantly (ie, the top and second to the right panel: beta = 2, the region for *α*_E_ > 1 and *α*_TVC_ < 0.75).
2. Landmark method: using the median TVC time, bias is minimal when $\beta_{\text{true}}=0$. However, for $\beta_{\text{true}}<0$, an upward bias is observed ($\hat{\beta }>\beta_{\text{true}}$, blue color), and for $\beta_{\text{true}}>0$, a downward bias is observed ($\hat{\beta }<\beta_{\text{true}}$, orange color).
3. TD Cox model: consistently exhibits near-zero bias across all conditions, reflecting robustness of this method.

In summary, contour plots clearly illustrate when each method's biases become more pronounced. We emphasize again that even in situations where bias is minimal, TD Cox models should be used.

### Estimation of Weibull Parameters With TVC in Survival Analysis Using the Maximum Likelihood Approach

The Data Supplement includes the details of how we developed the novel algorithm that can precisely estimate the parameters (${\hat{a}}_{E},{\hat{l}}_{E},{\hat{a}}_{\text{TVC}},{\hat{l}}_{\text{TVC}}$) and log HR ${\hat{\beta }}_{\text{true}}$ using the maximum likelihood approach.

In our simulations, given the Weibull distribution parameters for time to event (*α*_E_, *λ*_E_) and time to TVC (*α*_TVC_, *λ*_TVC_), we were able to simulate both the outcome survival time and time of TVC status change.^3^ As discussed above, if the *α*_E_ is relatively large and *α*_TVC_ is relatively small, the bias from the naïve Cox model will be subtle and might not affect the conclusion. However, in real-world applications, this information remains elusive. To address this challenge, we introduced a novel algorithm that can precisely estimate the parameters (${\hat{a}}_{E},{\hat{l}}_{E},{\hat{a}}_{\text{TVC}},{\hat{l}}_{\text{TVC}}$) and log HR ${\hat{\beta }}_{\text{true}}$ from real data (not simulated data) via the maximum likelihood approach (assuming that both time to outcome event and time to TVC follow Weibull distributions).

This task is intricate. The time to TVC might be censored either at the time of the event or at an independent censoring moment, causing the observed TVC time or event time no longer follow a Weibull distribution. In our maximum likelihood approach, we construct the full likelihood (as opposed to the partial likelihood in the TD Cox model) with five parameters (*α*_E_, *λ*_E,_ *α*_TVC_, *λ*_TVC_, $\beta_{\text{true}})$. Here is a brief workflow, and the detailed maximum likelihood is included in the Data Supplement.

1. Let A denote the time to TVC which follows a Weibull distribution with rate *λ*_TVC_ and shape *α*_TVC_, such that
  $$\mathrm{Pr}\left(A=a\right)=\alpha_{\text{TVC}}{\lambda_{\text{TVC}}}^{\alpha_{\text{TVC}}}a^{\alpha_{\text{TVC}}-1}\exp\left(-{{\lambda_{\text{TVC}}}^{\alpha_{\text{TVC}}}a}^{\alpha_{\text{TVC}}}\right)$$
2. Let T denote the time to event which follows a Weibull proportional hazard model,
  $$\lambda_{T}\left(t|A\right)=\lambda_{0}\left(t\right)\exp\left\{\beta I\left(t\geq A\right)\right\}$$
  where $\lambda_{0}\left(t\right)$ is the baseline Weibull hazard function $\lambda_{0}\left(t\right)=a_{E}t^{a_{E}-1}{l_{E}}^{a_{E}}$, such that an individual with A=∞ will have T following a Weibull distribution with rate *λ*_E_ and shape *α*_E._ Then, the distribution of the event time T given the TVC time A=a is given by
  $$\mathrm{Pr}\left(T=t│A=a\right)=\lambda_{T}\;\left(t│A=a\right)\;\;\exp{\{-\int }_{0}^{t}\;\lambda_{T}\;\left(s│A=a\right)ds\}$$
  $$=I\left(a\geq t\right)\lambda_{0}\left(t\right)\exp\left\{-\Lambda_{0}\left(t\right)\right\}+I\left(a<t\right)\lambda_{0}\left(t\right)\exp\left(\beta \right)\exp\left\{-\Lambda_{0}\left(a\right)-\left(\Lambda_{0}\left(t\right)-\Lambda_{0}\left(a\right)\right)\exp\left(\beta \right)\right\}.$$
3. Given a random sample of N individuals with observed data {$Y_{i},\Delta_{i},A_{i}I\left(A_{i}\leq Y_{i}\right)$} and assuming independent censoring such that the censoring time $C_{i}$ is independent with {${T_{i},A}_{i}$}, the likelihood function can be derived under different scenarios.
  - For an individual with $A_{i}\leq Y_{i}$ and ${\;\Delta }_{i}=1$, we have $T_{i}\leq C_{i}$. The likelihood is given by
    $$\mathrm{Pr}\left(A=A_{i}\right)\mathrm{Pr}\left(T=Y_{i}|A=A_{i}\right)\mathrm{Pr}\left(C\geq Y_{i}\right)$$
  - For an individual with $A_{i}\leq Y_{i}$ and ${\;\Delta }_{i}=0$, we have $T_{i}>\;C_{i}$. The likelihood is given by
    $$\mathrm{Pr}\left(A=A_{i}\right)\mathrm{Pr}\left(T>Y_{i}|A=A_{i}\right)\mathrm{Pr}\left(C=Y_{i}\right)$$
  - For an individual with $A_{i}>\;Y_{i}$ and ${\;\Delta }_{i}=1$, we have $T_{i}\leq C_{i}$. The likelihood is given by
    $$\int_{A_{i}>\;Y_{i}}\mathrm{Pr}\left(A=A_{i}\right)\mathrm{Pr}\left(T=Y_{i}|A=A_{i}\right)\mathrm{Pr}\left(C\geq Y_{i}\right)\;dA_{i}$$
  - For an individual with $A_{i}>\;Y_{i}$ and ${\;\Delta }_{i}=0$, we have $T_{i}>\;C_{i}$. The likelihood is given by
    $$\int_{A_{i}>\;Y_{i}}\mathrm{Pr}\left(A=A_{i}\right)\mathrm{Pr}\left(T>Y_{i}|A=A_{i}\right)\mathrm{Pr}\left(C=Y_{i}\right)\;dA_{i}$$
    Using the nleqslv R package, we obtain numerical estimates from nonlinear equations using the Newton method. Comprehensive details are provided in the Data Supplement, and this algorithm is also integrated into our R Shiny tool TVCurve. As demonstrated in the Data Supplement (Fig S7), our algorithm accurately estimates the Weibull parameters (*α*_E_, *λ*_E,_ *α*_TVC_, *λ*_TVC_) and the log HR $\beta_{\text{true}}$ (Data Supplement, Figs S7a-S7e). The TD Cox model also accurately estimates $\beta_{\text{true}}$ (Data Supplement, Fig S7f) but cannot estimate other parameters given its semiparametric nature.
